# The Rating of Voluntary Hospitals

**Published:** 1897-09-25

**Authors:** Mary Wardell


### Editor's Letter-Box.

[Our Correspondents are reminded that prolixity is a great bar to publication, and that brevity of style and conciseness of statement greatly facilitate early insertion.]

### THE RATING OF VOLUNTARY HOSPITALS.

Miss Mary Wardell writes: I am exceedingly glad to see in your issue of September 11th a paper on "Rating of Voluntary Hospitals." I have for long been feeling very sore on this subject. The amount I have to pay for rates on this comparatively small voluntary institution presses very hardly on me; the more so as I do not benefit by the drainage, lighting, &c., on which most of the money is expended. No system of sewers comes near my home, and I have had to lay out a costly system of sub-soil irrigation drainage, carried out and supervised by Mr. Rogers Field, C.E. The only light on the road near my home is that afforded by a light over the gate, placed by myself—an oil lamp. My home is rated at the value of £283 15s., which is far too high. If to be let or sold we should never receive what we have spent on it, to fit it for its special work. I have this year paid £92 4s. 6d. in rates. I have twice appealed personally to the Board, besides writing a letter to each of the guardians—39 in all, I think. If anything can be done to reduce this oppressive taxation of a charitable work, I shall be thankful, and if I can forward such a movement in any way I would be willing to join in any protest or petition to Parliament, or to any other influential body.

